# CFTR Knockdown induces proinflammatory changes in intestinal epithelial cells

**DOI:** 10.1186/s12950-015-0107-y

**Published:** 2015-11-07

**Authors:** Karoline St-Martin Crites, Geneviève Morin, Valérie Orlando, Natacha Patey, Catherine Cantin, Judith Martel, Emmanuelle Brochiero, Geneviève Mailhot

**Affiliations:** Research Centre, CHU Sainte-Justine, 3175 Cote Sainte-Catherine Rd, Montreal, Quebec H3T 1C5 Canada; Research Center, CHUM, 900 Saint-Denis Street, Montreal, Quebec H2X 0A9 Canada; Department of Medicine, Université de Montreal, 2900, Édouard-Montpetit Blvd, Montreal, Quebec H3T 1J4 Canada; Department of Nutrition, Université de Montreal, 2405 Cote Sainte-Catherine Rd, Montreal, Quebec H3T 1A8 Canada

**Keywords:** CFTR, Inflammation, Intestinal cell line, Cystic fibrosis

## Abstract

**Background:**

Hyperinflammation is a hallmark feature of cystic fibrosis (CF) airways. However, inflammation has also been documented systemically and, more recently, in extrapulmonary CF-affected tissues such as the pancreas and intestine. The pathogenesis of CF-related inflammation and more specifically the role of the cystic fibrosis transmembrane conductance regulator (CFTR) in that respect are not entirely understood. We have tested the hypothesis that genetic depletion of CFTR will affect the inflammatory status of human intestinal epithelial cell lines.

**Methods:**

CFTR expression was genetically depleted from Caco-2/15 and HT-29 cells using short hairpin RNA interference (shRNAi). Inflammatory conditions were induced by the addition of human recombinant tumor necrosis factor (TNF) or Interleukin-1β (IL-1β) for various periods of time. Gene expression, mRNA stability and secreted levels of interleukin (IL)-6, −8 and 10 were assessed. Analysis of pro- and anti-inflammatory signaling pathways including mitogen-activated protein kinases (p38, ERK 1/2 and JNK), nuclear factor of kappa light polypeptide gene enhancer in B-cells inhibitor alpha (IκBα), and nuclear factor-kappa B (NF-κB) was also performed. Eosinophils were counted in the jejunal mucosa of *Cftr−/−* and *Cftr+/+* mice.

**Results:**

CFTR gene and protein knockdown caused a significant increase in basal secretion of IL-8 as well as in IL-1β-induced secretion of IL-6 and −8. Release of the anti-inflammatory cytokine, IL-10, remained unaffected by CFTR depletion. The enhanced secretion of IL-8 stems in part from increased IL8 mRNA levels and greater activation of ERK1/2 MAPK, IκBα and NF-κB in the CFTR knockdown cells. By contrast, phosphorylation levels of p38 and JNK MAPK did not differ between control and knockdown cells. We also found a higher number of infiltrating eosinophils in the jejunal mucosa of *Cftr* −/− females, but not males, compared to *Cftr* +/+ mice, thus providing *in vivo* support to our *in vitro* findings.

**Conclusion:**

Collectively, these data underscore the role played by CFTR in regulating the intestinal inflammatory responses. Such findings lend support to the theory that CFTR exerts functions that may go beyond its role as a chloride channel whereby its disruption may prevent cells to optimally respond to exogenous or endogenous challenges. These observations are of particular interest to CF patients who were found to display alterations in their intestinal microbiota, thus predisposing them to pathogens that may elicit exaggerated inflammatory responses.

**Electronic supplementary material:**

The online version of this article (doi:10.1186/s12950-015-0107-y) contains supplementary material, which is available to authorized users.

## Background

Cystic fibrosis (CF) is the most prevalent life-shortening genetic disease among Caucasians striking approximately one in 3500 newborns [1]. Fortunately, CF is no longer a pediatric disease due to advances in therapeutics, whereby consequently CF patients are now likely to live into their late thirties and beyond. CF is caused by mutations in the gene encoding the chloride channel “Cystic fibrosis transmembrane conductance regulator” (CFTR). The CFTR dysfunction, along with other contributing factors, leads to a wide array of clinical symptoms affecting predominantly the lungs and gastrointestinal system.

Chronic inflammation is a hallmark feature of CF affecting mainly the lungs but has also been documented both systemically [2] and in extra-pulmonary tissues [3–5]. The origin of inflammation in CF-affected organs has been a topic of controversy as it is yet unclear whether it is of primary or secondary origin. In airway cells, it has been reported that defective CFTR leads to impaired immune cell functions and exaggerated proinflammatory responses [6, 7] whereas others have suggested that inflammation occurs secondary to bacterial infection [8]. Similar to the airways, CFTR is highly expressed in the epithelial cells of the small and large intestine, with the greatest levels found in the duodenum [9]. Another common feature between the airways and intestinal tract is that they are both constitutively exposed to significantly large amounts of bacteria and bacterial products, which may be responsible for the activation of inflammation. Lloyd-Still was among the first to report that CF patients were more predisposed to inflammatory bowel diseases with a prevalence of 7 times that of controls mostly accounted for by Crohn’s disease, which was found to be 17 times more prevalent in CF patients [10]. Intestinal inflammation has since then been documented in CF children and young adults [5, 11, 12]. Smyth *et al.* have studied intestinal inflammation in pancreatic-insufficient CF children and found increased levels of inflammatory cytokines, immunoglobulins and other proteins in whole gut lavage [11]. Immunohistochemical comparison of duodenal biopsies from pancreatic-insufficient CF patients and healthy controls revealed an increased infiltration of mononuclear cells expressing the intercellular Adhesion Molecule 1 (ICAM-1), CD-25, Interleukin (IL)-2 and Interferon γ (IFNγ) in the lamina propria of CF patients [5]. More recently, Werlin et al. used wireless capsule enteroscopy to document intestinal mucosal abnormalities in a large proportion of CF patients and reported high fecal calprotectin levels suggestive of intestinal inflammation [12]. A comparison of CF children to healthy controls and children with Crohn’s disease showed that CF intestinal inflammation is distinct from that seen in patients with Crohn’s disease and is characterized by elevated calprotectin but normal levels of the biomarkers S100A12 and osteoprotegerin [13]. Despite such evidence, little is known regarding the pathogenesis of CF intestinal inflammation, which has been attributed to numerous factors including chronic enzyme usage, dysmotility, and bacterial overgrowth. However, pancreatic sufficient patients also exhibited morphological small bowel changes thereby suggesting that intestinal inflammation may be intrinsically related to CF [12]. Interestingly, small intestinal inflammation was not observed in subjects with non-CF pancreatic insufficiency, suggesting that pancreatic insufficiency itself is unlikely a contributing factor to intestinal inflammation [5]. Additionally, the demonstration of intestinal inflammation in a CF mice model, in the overt absence of lung disease, chronic infections, pancreatic insufficiency and pancreatic enzyme replacement therapy (PERT), provides additional support for the role of CFTR dysfunction in that respect [4].

In order to distinguish the role of CFTR from that of other external factors in the development of intestinal inflammation, we investigated whether manipulation of CFTR expression and function influences the inflammatory profile of intestinal cells *in vitro* under pathogen-free conditions. Here, we documented that CFTR knockdown of two intestinal epithelial cell lines, Caco-2/15 and HT-29, induced changes in the inflammatory response system as evidenced by an increase in gene expression and secretion of IL-6 and −8, as well as a greater activation of the ERK1/2 MAPK, IκBα and NF-κB pathways.

## Methods

### Materials

Eagle’s minimum essential medium, McCoy’s 5a medium, fetal bovine serum (FBS), non-essential amino acids (NEAA), penicillin-streptomycin (PS), phosphate buffered saline (PBS), bovine serum albumin (BSA) and puromycin were obtained from Wisent (St-Bruno, Qc, Canada). Short hairpin RNAs (shRNAs) targeting CFTR and individually cloned into plko.1-puromycin vector were purchased from Open Biosystems (Huntsville, AL). A lentiviral negative control, pLKO.1-scrambled, was purchased from Addgene (Cambridge, MA). Hexadimethrine bromide (Polybrene), forskolin, 3-Isobutyl-1-methylxanthin (IBMX), actinomycin D, Triton X-100 and paraformaldehyde came from from Sigma (St-Louis, MO). Recombinant human IL-1β and TNF were from PeproTech (Quebec, Canada). Trizol and M-MLV reverse transcriptase were from Invitrogen (Carlsbad, CA). The 1X SsoFast EvaGreen Supermix with Low ROX and the protein assay kit were from Bio-Rad (Hercules, CA). M-PER™ mammalian protein extraction buffer was from Thermo Scientific (Rockford, IL). Antibodies used were from the following suppliers: anti-CFTR and phosphorylated and total anti-p38, anti-Extracellular signal-regulated kinases (ERK) 1/2, anti-c-Jun N-terminal kinases (JNK) and anti-nuclear factor of kappa light polypeptide gene enhancer in B-cells inhibitor alpha (IκBα) were from Cell Signaling Technology (Beverly, MA), anti-Bcl-2-associated X protein (Bax) and anti-NF-κB p65 were from Santa Cruz Biotechnology Inc. (Dallas, TX), anti-β-actin was from Sigma and species-specific horseradish peroxidase (HRP)-conjugated secondary antibodies were from Bio-Rad. Alexa Fluor® 594 donkey anti-goat (1:1000) and the ProLong® Gold antifade reagent with 4′,6-diamidino-2-phenylindole (DAPI) were from Life Technologies Inc. (ON, Canada). The Amersham™ ECL™ Prime Western Blotting Detection Reagent was from GE Lifesciences (Baie d’Urfe, QC, Canada). Western-blot stripping buffer was from ZmTech Scientifique (Montreal, QC, Canada).

### Intestinal cell lines

The human colon carcinoma cell line, Caco-2/15, a stable clone from the parent Caco-2 cells (American Type Culture Collection, Rockville, MD), was obtained from Dr. Emile Levy (CHU Sainte-Justine Research Center, Montreal, Quebec, Canada). HT-29 cells, a human colon carcinoma cell line, were purchased from ATCC®. Although of cancerous origin, these cells are widely used for the study of non-malignant epithelial intestinal physiology. Caco-2/15 were used for all experiments and maintained at subconfluent stages in EMEM supplemented with 5 % FBS, 1 % NEAA and 1 % PS. All experiments were carried out on cells from passage 26 to 31. HT-29 cells were maintained at subconfluent stages in McCoy’s 5a supplemented with 10 % FBS and 1 % PS and specific experiments were carried out on cells from passage 7 to 9. Cells were seeded onto 12-well plates at a density of 2 x 10^5^ cells/well after a trypan blue exclusion test to assess cell survival. Cells were cultured for a period of three to four days prior to any experimentation.

### Lentivirus production and cell infection

Lentiviral stocks were prepared with the use of HEK293FT as packaging cells according to the method described previously [14, 15]. The viral supernatants were collected 2 days after, and lentiviruses concentrated following a fast ultrafiltration with Amicon® Ultra-15 centrifugal filter device (EMD Millipore, Bellerica, MA). Concentrated lentiviruses were aliquoted and stored at −80 °C until use.

Caco-2/15 and HT-29 cells were infected with specific lentivirus in the presence of Polybrene as published previously [14, 15]. When cells reached 80 % confluence, they were plated and allowed to proliferate in the presence of puromycin to select for cells showing stable integration of the shRNA constructs. Infection efficiency was assessed by measuring CFTR gene and protein expression by quantitative PCR (Q-PCR) and Western Blotting. Cells infected with lentivirus containing scrambled shRNA sequence served as controls since preliminary experiments had shown comparable cell viability and integrity as well as gene and protein CFTR expression between scrambled and non-infected cells [14].

### Pharmacological activation of CFTR

To activate CFTR, cells were treated 24 h with a cAMP-increasing cocktail made of 10 μM forskolin and 100 μM 3-Isobutyl-1-methylxanthin (IBMX) in the absence of serum.

### Proinflammatory challenge and cell viability

To induce a proinflammatory state, subconfluent (80–90 % of confluence) cells, corresponding to homogenously undifferentiated cells, were stimulated with 10 or 25 ng/mL of recombinant human IL-1β or TNF for times ranging from 5 minutes to 24 h. Trypan blue staining and cell counting were performed to determine whether the compounds had any effect on cell viability. All experiments were carried out in the absence of serum except for experiments with HT-29 cells, as these cells exhibited high cell mortality in the absence of serum.

### Interleukin-6, −8 and −10 secretion

Cell culture supernatants were collected, centrifuged for 5 min at 13 000 rpm at 4 °C to remove cell debris and stored at −80 °C until they were assayed for IL-6, −8 and −10 by ELISA (OptEIA antibody set; BD Biosciences, ON, Canada), according to the manufacturer’s instructions. Absorbances were measured at 450 nm wavelength using the Spectra RainBow plate reader (Tecan Systems inc., San Jose, CA). Cellular protein concentration was determined using the Bio-Rad protein assay kit and concentration of interleukins normalized to total protein content.

### RNA isolation, RT-PCR and Q-PCR

RNA was isolated from cells using Trizol according to manufacturer’s instructions. Two μg of total RNA was reverse transcribed by using the M-MLV reverse transcriptase. CFTR and IL-8 mRNA expressions were determined by Q-PCR. Briefly, 15 μl Q-PCR reaction containing cDNA diluted 100 times, 1X SsoFast EvaGreen Supermix with Low ROX and 0.5 μM of CFTR or IL-8 specific and intron-spanning primers was performed using the Mx3000p Q-PCR System (Agilent Technologies, Santa Clara, CA) under the following conditions: 95 °C for 30 s, 45 cycles of 95 °C for 5 s, 60 °C for 30 s, and 72 °C for 20 s. Each reaction was performed in duplicate. The relative quantification of both genes was normalized to the 60S ribosomal protein L27 (RPL27) reference gene and fold-induction was determined from the average threshold cycle (Ct) using the standard curve method. PCR efficiency was calculated from the slope of the standard curve, and generated using a five-fold dilution series of cDNA template obtained from IL-1β-treated cells.

### IL-8 mRNA stability

IL-8 mRNA stability was assessed by decay after the addition of actinomycin D. Briefly, CFTR knockdown and control cells were treated first, as detailed above, then transcription was blocked by the addition of 5 μg/mL of actinomycin D. Cells were collected, at times ranging from 2 to 8 h after actinomycin treatment and total RNA extracted using Trizol. Q-PCR for IL-8 was carried out as described and IL-8 mRNA levels were normalized to RPL27, whose expression remained unaffected by actinomycin treatment.

### Immunoblotting

Cells were washed twice with cold PBS 1X, scraped, and lysed with cold M-PER™ mammalian protein extraction buffer. Cell lysates were pelleted through centrifugation at 13 000 rpm at 4 °C for 5 min. Lysates were stored at −20 °C for later use. Protein concentration was determined using the Bio-Rad protein assay kit. Briefly, between 15 to 40 ug of total protein were separated on 10 % SDS-PAGE and transblotted onto polyvinylidene difluoride (PVDF) membranes (GE Lifesciences). After blocking for 1 h in a TBS solution mixed with 5 % nonfat dehydrated milk, membranes were blotted overnight at 4 °C with anti-CFTR (1:1000), or phosphorylated anti-p38, anti-ERK 1/2, anti-JNK, anti- IκBα and anti-Bax (all 1:1000) followed by a 1 h-incubation with species-specific horseradish peroxidase (HRP)-conjugated secondary antibodies (1:10,000). Visualization of the protein bands on X-ray film (Bioflex MSI film; Ultident, St-Laurent, QC, Canada) was achieved with the Amersham™ ECL™ Prime Western Blotting Detection Reagent. In some cases, membranes were stripped with a western-blot stripping buffer according to the manufacturer’s instructions, and reprobed overnight at 4 °C with anti-β-actin (1:5000), or with total p38, ERK 1/2, JNK, and IκBα (1:1000). Before protein visualization, membranes underwent a second 1 h-incubation with species-specific HRP-conjugated secondary antibodies (1:20,000). The films were quantified by computer-assisted scanning densitometry using UN-SCAN-IT software (Silk Scientific, Orem, UT).

### NF-κB immunofluorescence

To determine the expression of NF-κB, 4 x 10^4^ Caco-2/15 cells were grown on glass Lab-Tek®II chamber slides™ (Fisher Scientific, ON, Canada) 2 to 3 days before experiments. Cells were then washed with PBS 1X and fixed with paraformaldehyde 3.7 % at room temperature for 10 min. After two PBS washes, cells were permeabilized with Triton 0.1 % at room temperature for 10 min and washed again with PBS. Cells were then blocked with a BSA 1 % solution at room temperature for 30 min before being labeled with a goat anti-human NF-κB p65 antibody (1:200) for 1 h at room temperature. After 3 washes with the blocking solution, cells were incubated with an Alexa Fluor® 594 donkey anti-goat IgG (1:1000) for 1 h at room temperature. Cells were then washed 3 times with the blocking solution and then twice again with PBS after which the media chambers were removed. Slides were mounted with the ProLong® Gold antifade reagent with DAPI for nucleus staining and the immunofluorescence was detected using a fluorescent microscope (Leica Microsystems Inc., ON, Canada). The level of fluorescence in a given region (nucleus, whole cell and background) was quantified with the Image J program using the area, the integrated density and mean gray value readings. Corrected total cell fluorescence (CTCF) was calculated using the following equation: CTCF = Integrated Density – (Area of selected region x Mean gray value of background reading). The cytoplasmic CTCF was obtained after subtracting the nucleus CTCF from its whole corresponding cell CTCF.

### Mice

The *Cftr−/−* and *Cftr +/+* mice on a BALB/c background were initially provided by Dr. Christina Haston from McGill University, Montreal, Canada. All mice were bred and housed in the CHU Sainte-Justine Research Institute animal facility. To avoid risk of intestinal obstruction and premature death, all *Cftr −/−* mice were fed *ad libitum* standard chow diet moistened with reverse osmotic water and 17.8 mmol/L polyethylene glycol 3350 (PEGLYTE™; Pharmascience, Quebec, Canada) in their drinking water from three weeks of age until euthanasia. To eliminate the confounding effect of diet on intestinal physiology, control mice were also maintained on the same feeding and drinking regimen. These regimens did not alter food and water consumption of the mice. After weaning, *Cftr−/−* and *Cftr +/+* mice were grouped based on their sex and were co-housed. Mice were euthanized at 12 weeks of age by intracardiac puncture under anesthesia. All procedures were in accordance with the CHU Sainte-Justine Research Institute Animal Care committee.

### Histology

At sacrifice, the entire intestinal tract was collected and inspected to detect the presence of intestinal obstruction; none of the mice had visual evidence of such obstruction. The entire jejunum, which corresponds roughly to the second third of the small intestine, was removed, longitudinally opened, gently flushed with ice-cold PBS and cleared of feces. The specimens were fixed with buffered-formalin phosphate 10 %, paraffin-4 μm sections were prepared, mounted on microscope slides, and stained with hematoxylin eosin safran. Eosinophils were quantified in intercryptic lamina propria by counting at 400 X magnification the number of cells in ten randomly selected fields. Female and male mice were analysed separately by a pathologist blinded to the genotype and sex of the mice.

### Statistical analysis

Data are expressed as means ± SEM. Comparison of means was achieved using analysis of variance, unpaired Student’s *t* or nonparametric Mann–Whitney tests where appropriate. All statistical analyses were conducted with SPSS 21 software. Statistical significance was set at *p* <0.05.

## Results

### Cell viability and apoptosis

Caco-2/15 cell viability was first assessed in order to exclude any detrimental effect of the experimental conditions. Neither lentiviral infection nor treatment with TNF, or IL-1β, significantly affected cell viability compared to untreated cells (see Additional file 1). Given that the trypan blue exclusion assay cannot distinguish between necrosis and apoptosis, we also measured the expression of the pro-apoptotic protein Bax by Western blot. Neither lentiviral infection nor treatment with TNF, or IL-1β, significantly affected Bax protein expression in Caco-2/15 cells (see Additional file 2).

### Secretion of interleukins

**Fig. 1:**
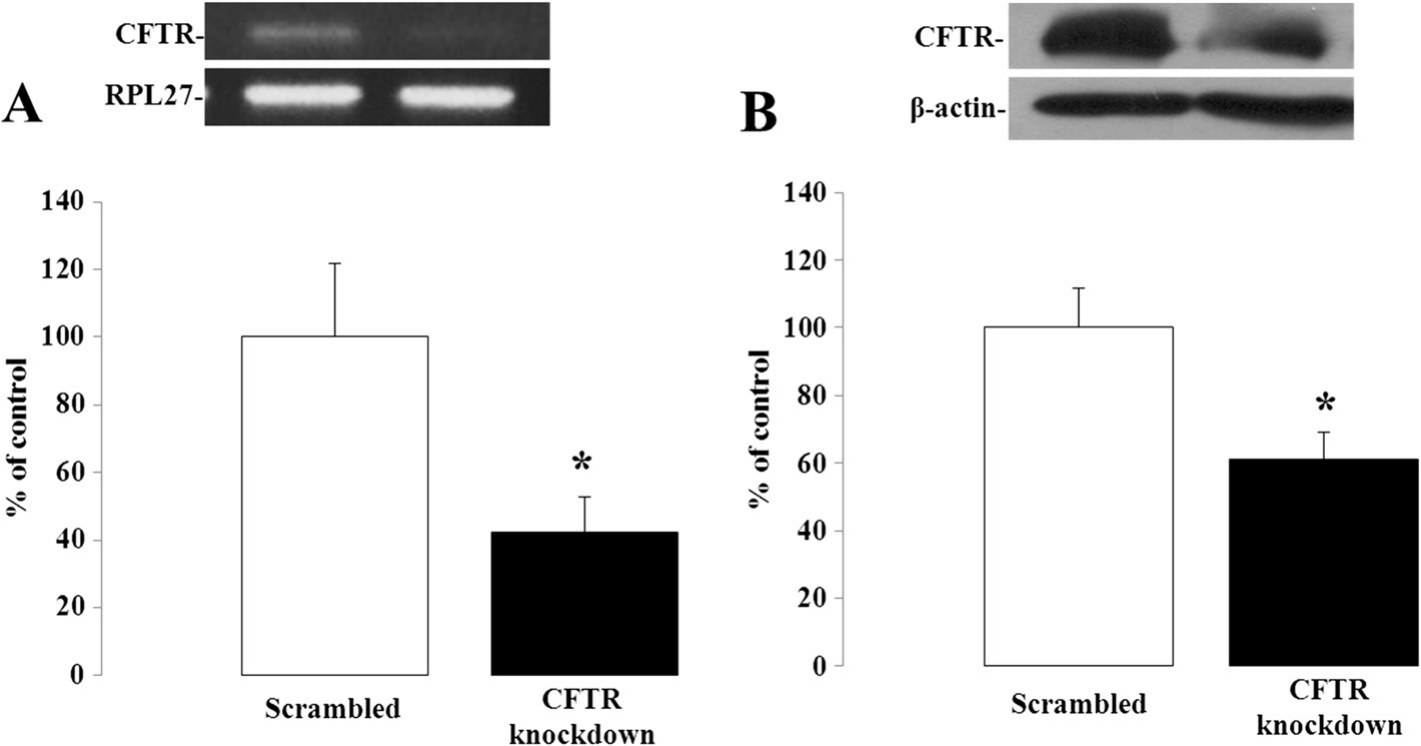
CFTR knockdown in Caco-2/15 cells. Caco-2/15 cells were infected with a lentiviral vector carrying either a scrambled sequence or shRNAi against CFTR and analyzed for gene (**a**) and protein (**b**) expression of CFTR by Q-PCR and Western blotting when they reached 80 to 90 % of confluence. Results represent the means ± SEM of independent experiments and are illustrated as % of controls after calculating the data as densitometric ratios of CFTR to the housekeeping gene RPL27 for gene expression (*n* = 10) or CFTR to β-actin for protein expression (*n* = 4). *p < 0.05 vs. scrambled-infected cells

We used the shRNAi experimental approach to genetically deplete CFTR in Caco-2/15 intestinal cells. Lentiviral infection of Caco-2/15 cells, with the same vector carrying a scrambled sequence, neither affected CFTR mRNA (100 ± 24.9 vs. 98 ± 13.7 %, *n* = 4–16) nor protein expression (100 ± 11.5 vs. 99 ± 11.5 %, *n* = 4) compared to non-infected cells (data not shown). In contrast, infection of Caco-2/15 cells with a lentiviral vector carrying shRNAi against CFTR resulted in a 52 and 39 % reduction in CFTR gene and protein expression respectively when compared to scrambled-infected cells (Fig. 1).

**Fig. 2:**
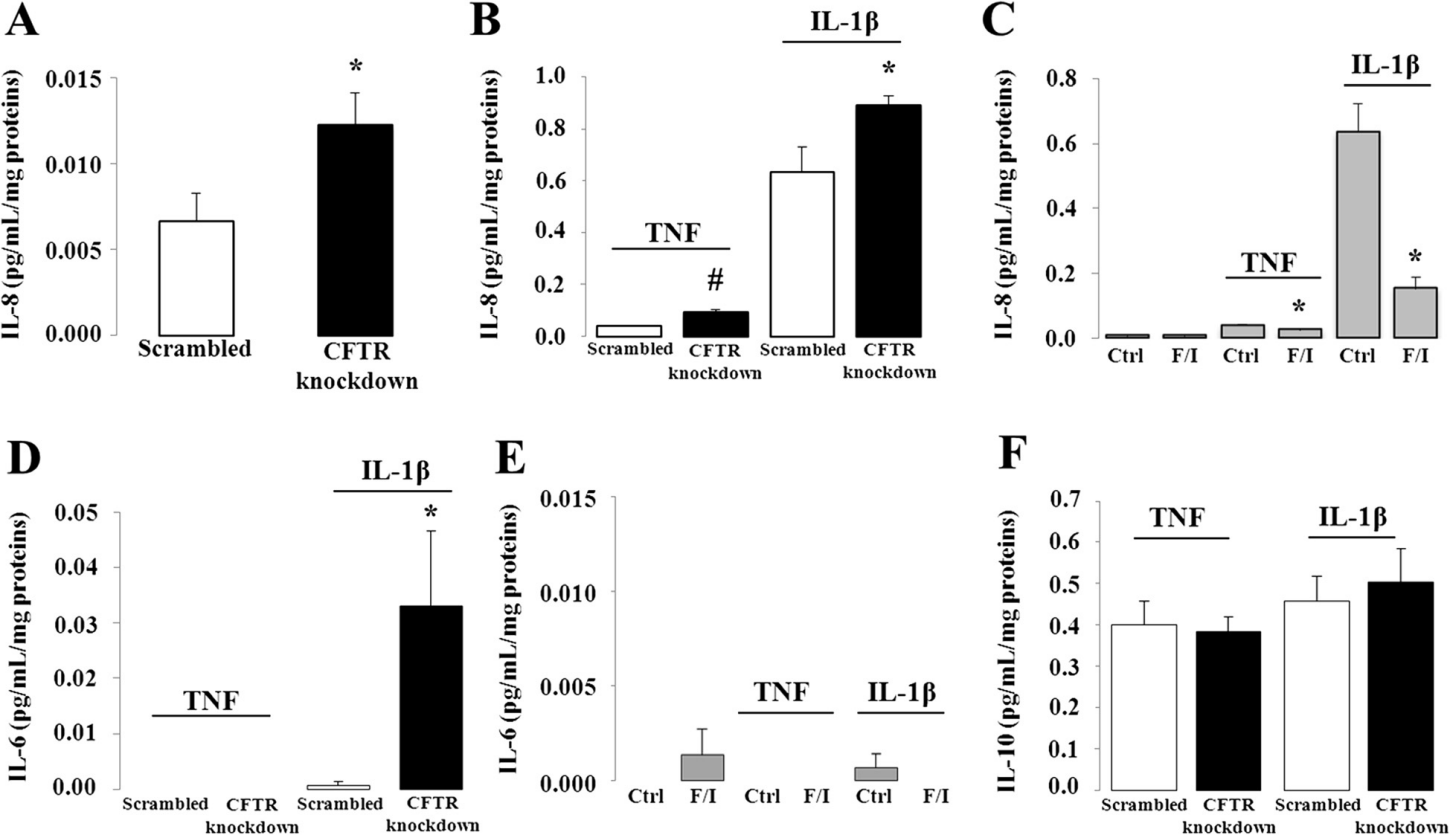
Effect of CFTR knockdown and activation on IL-6, −8 and −10 secretion in Caco-2/15 cells. CFTR knockdown or control (Scrambled) cells were incubated 24 h in the absence (**a**) or presence (**b**, **d**, **f**) of 25 ng/mL of either TNF or IL1-β. (**c**, **e**) TNF or IL1-β-stimulated interleukin secretion following pharmacological activation of CFTR. Prior to incubation with TNF or IL1-β, cells with intact CFTR were treated with a mixture of 10 μM forskolin and 100 μM IBMX for 24 h. Supernatants were then collected to quantify the secretion of IL-8 (**a**-**c**), −6 (**d**-**e**) and −10 (**f**) by ELISA. Results represent the means ± SEM of *n* = 3–8 independent experiments and are illustrated as pg/mL normalized to total protein concentration. *p <0.05 and #*p* <0.0001 vs. scrambled-infected cells

To rule out any non-specific effect of the scrambled sequence, IL-8 secretion was first compared between non-infected and scrambled-infected cells. Both basal (0.01 ± 0.001 vs. 0.01 ± 0.001 pg/mL, *n* = 4) and IL-1β-stimulated (1.03 ± 0.20 vs. 1.11 ± 0.24 pg/mL, *n* = 4) IL-8 secretion were similar between the two conditions. Therefore, for all subsequent experiments, scrambled-infected cells were used as controls. IL-8 basal secretion was moderately but significantly increased in CFTR-depleted cells compared to controls (Fig. 2a) whereas scrambled and CFTR knockdown cells released undetectable levels of IL-6 at baseline (data not shown). To test the effects of CFTR knockdown on IL-6 and −8 production in response to pro-inflammatory cytokines, the cells were incubated for 24 h in the presence of either TNF or IL-1β. To account for potential differences in cell number and viability, interleukin concentrations were normalized to total protein content. Both cytokines caused an increase in IL-8 secretion with respect to basal levels confirming the induction of a pro-inflammatory state whereas IL-6 secretion was only observed in response to IL-1β, although the amount released was much lower. In either case, CFTR gene depletion led to a significant enhancement of their secretion (Fig. 2b and d). The secretion of IL-6 and-8 was respectively 33 and 1.4-fold higher in IL-1β-treated knockdown cells compared to controls. While IL-8 secretion was 2.4- fold greater in knockdown cells treated with TNF than controls, this cytokine failed to induce the release of IL-6. Conversely, CFTR activation by means of a cAMP-increasing cocktail containing forskolin and IBMX resulted in a marked blunting of cytokine-induced IL-8 release, with a more pronounced effect in the IL-1β than the TNF-challenged cells (Fig. 2c). Given the nearly undetectable basal and cytokine-stimulated production of IL-6 in Caco-2/15 cells with intact CFTR, forskolin and IBMX exerted no further effect on the secretion of IL-6 (Fig. 2e). The observed proinflammatory changes in response to CFTR depletion prompted us to investigate whether it also modulates the release of the anti-inflammatory cytokine, IL-10. At baseline, the IL-10 concentrations were below the detection limit (<2 pg/ml) in cell supernatants from either scrambled or CFTR knockdown cells (data not shown). The addition of TNF or IL-1β induced similar IL-10 secretion irrespective of the level of expression of CFTR (Fig. 2f).

In order to distinguish the consequences of CFTR protein depletion from those of its pharmacological inhibition, we treated Caco-2/15 cells with two different CFTR inhibitors, CFTRinh-172 and GlyH-101. Given that these inhibitors have never been used in Caco-2/15 cells, short-circuit current measurements in an Ussing chamber were first undertaken to assess the impact of chronic exposure to GlyH-101 or CFTRinh-172 on chloride currents in order to determine the extent of the CFTR inhibition. We tested a wide range of inhibitor concentrations (5 to 20 μM) and durations of treatment (from 6 to 72 h with fresh inhibitor being replenished every 24 h). Surprisingly, we were unable to achieve a lasting inhibition of CFTR. At best, we achieved a 50 % inhibition of CFTR chloride current with the highest dose of CFTRinh-172 (20 μM) but at the expense of compromised cell viability (74 ± 1.5 %).

We then chose to focus on CFTR-depleted cells and sought to investigate the cellular mechanisms underlying the enhanced IL-8 secretion in CFTR knockdown intestinal cells, given that it was the most abundantly produced of the three cytokines examined.

### IL-8 steady-state mRNA levels and stability

**Fig. 3:**
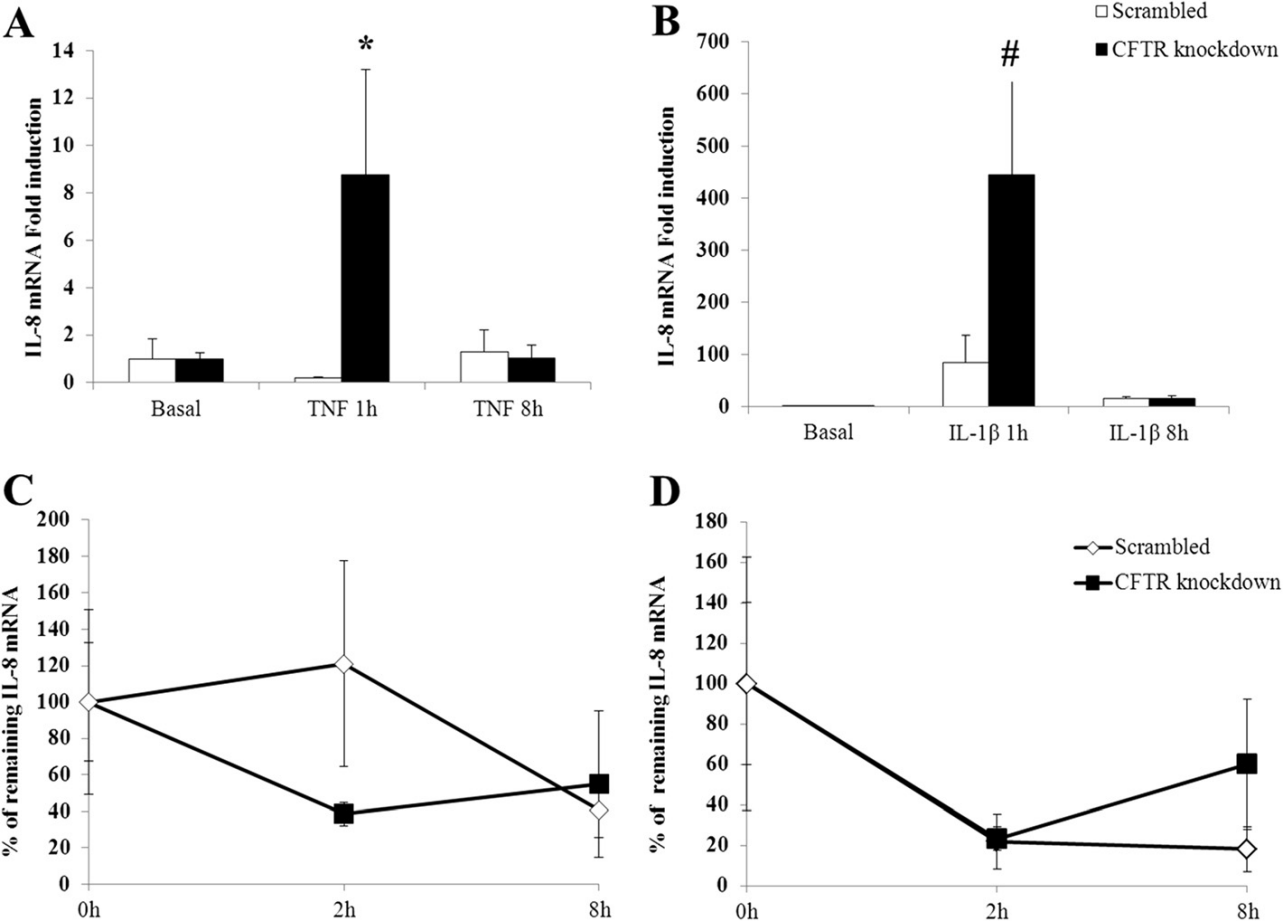
Effect of CFTR knockdown on IL-8 steady-state mRNA levels and IL-8 mRNA stability. CFTR knockdown or control (Scrambled) cells were incubated 1 and 8 h in the absence (**a**) or presence (**b**) of 25 ng/mL of either TNF or IL1-β. Total RNA was collected and subjected to Q-PCR using intron-spanning primers for the IL-8 gene. For mRNA stability, cells were incubated with 25 ng/mL of TNF (**c**) or IL1-β (**d**) for 1 h and then subjected to actinomycin D (5 μg/mL) to block transcription. Cells were incubated in the presence of actinomycin D for 2 and 8 h after which RNA was collected and analysed by Q-PCR for IL-8 mRNA expression. IL-8 mRNA was normalized to that of the housekeeping gene RPL27. Data represent the means ± SEM of *n* = 3-5 independent experiments. Steady state mRNA levels are reported as fold induction over basal levels. For mRNA stability, values represent percentages of remaining mRNA versus mRNA levels before the addition of actinomycin (time 0). *p <0.05 and #*p* <0.0001 vs. scrambled-infected cells

We first determined whether the increased IL-8 secretion in CFTR knockdown cells involved the upregulation of IL-8 gene expression and/or enhanced mRNA stability. Basal IL-8 mRNA levels did not differ between CFTR knockdown and scrambled-infected cells (Fig. 3a-b). However, CFTR gene depletion led to a significant enhancement of IL-8 mRNA expression after either TNF or IL-1β challenge compared to scrambled cells (Fig. 3). Notably, this effect was observed within 1 h of exposure to the cytokines whereby TNF, and IL-1β induced a 9 and 445-fold increase in IL-8 gene expression of knockdown cells as opposed to 0.2 and 84 fold in the scrambled cells. We then considered whether CFTR knockdown may have a post-transcriptional effect by increasing IL-8 mRNA stability. Therefore, scrambled and CFTR knockdown cells were subjected to actinomycin D pulse-chase, and as a result, CFTR knockdown did not increase mRNA stability of IL-8 upon TNF or IL-1β treatment (Fig. 3c-d).

### Pro-inflammatory signalling pathways

**Fig. 4:**
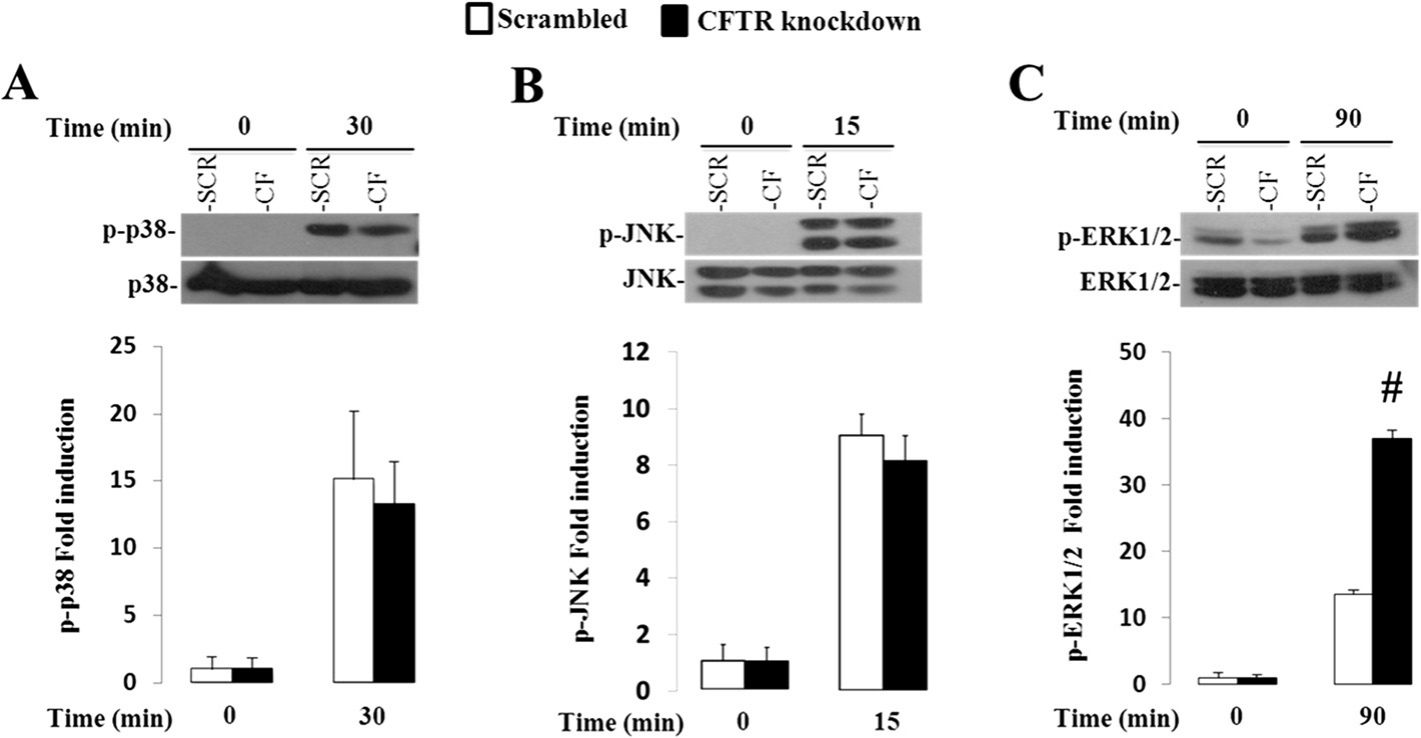
Effect of CFTR knockdown on p38MAPK, JNK, ERK1/2 pathways in Caco-2/15 cells. Cells were incubated from 0 to 120 min with 25 ng/mL of IL1-β. Protein expression of phosphorylated p38(**a**), JNK(**b**) and ERK1/2 MAPK(**c**) (p-p38, p-JNK and p-ERK1/2) and total p38, JNK and ERK1/2 MAPK was analyzed by Western blotting. Data represent the means ± SEM of *n* = 3-9 independent experiments and the phosphorylated form expression was normalized to that of total form and reported as fold induction over basal expression. #*p* <0.0001 vs. scrambled-infected cells

Cytokine-induced secretion of IL-8 is mediated by mitogen-activated protein kinases (MAPKs) [16]. Given that some of these pathways have been previously shown to be upregulated in CF-affected cells [17, 18], we decided to examine the activation of p38, ERK and JNK MAPK pathways in CFTR knockdown intestinal cells. Since IL-1β induced a much greater inflammatory response in Caco-2/15 cells than TNF, we deliberately focused on IL-1β stimulation of Caco-2/15 cells in all subsequent experiments. Incubation with IL-1β for a period of 15 to 60 min resulted in a significant activation of p38MAPK, which peaked within 30 min of stimulation. However, phosphorylated levels of p38MAPK in response to IL-1β were indistinguishable between CFTR knockdown and control cells (Fig. 4a). Similar findings were obtained with JNK phosphorylation, which increased within 15 min of IL-1β treatment, yet remained unaffected by CFTR gene depletion (Fig. 4b). Maximal ERK phosphorylation, on the other hand, occurred after 90 min of exposure to IL-1β and was enhanced by a 2.6-fold in CFTR knockdown cells. Indeed, IL-1β induced a 14-fold increase of ERK phosphorylation in scrambled cells, which nearly tripled (37-fold) in CFTR-depleted cells (Fig. 4c). IL-1β-stimulated ERK phosphorylation remained significantly greater in CFTR knockdown after 120 min (11 vs 31-fold, *p* < 0.001; data not shown). Of note, total protein levels of MAPKs remained unchanged by the experimental treatments.

### NF-κB

**Fig. 5:**
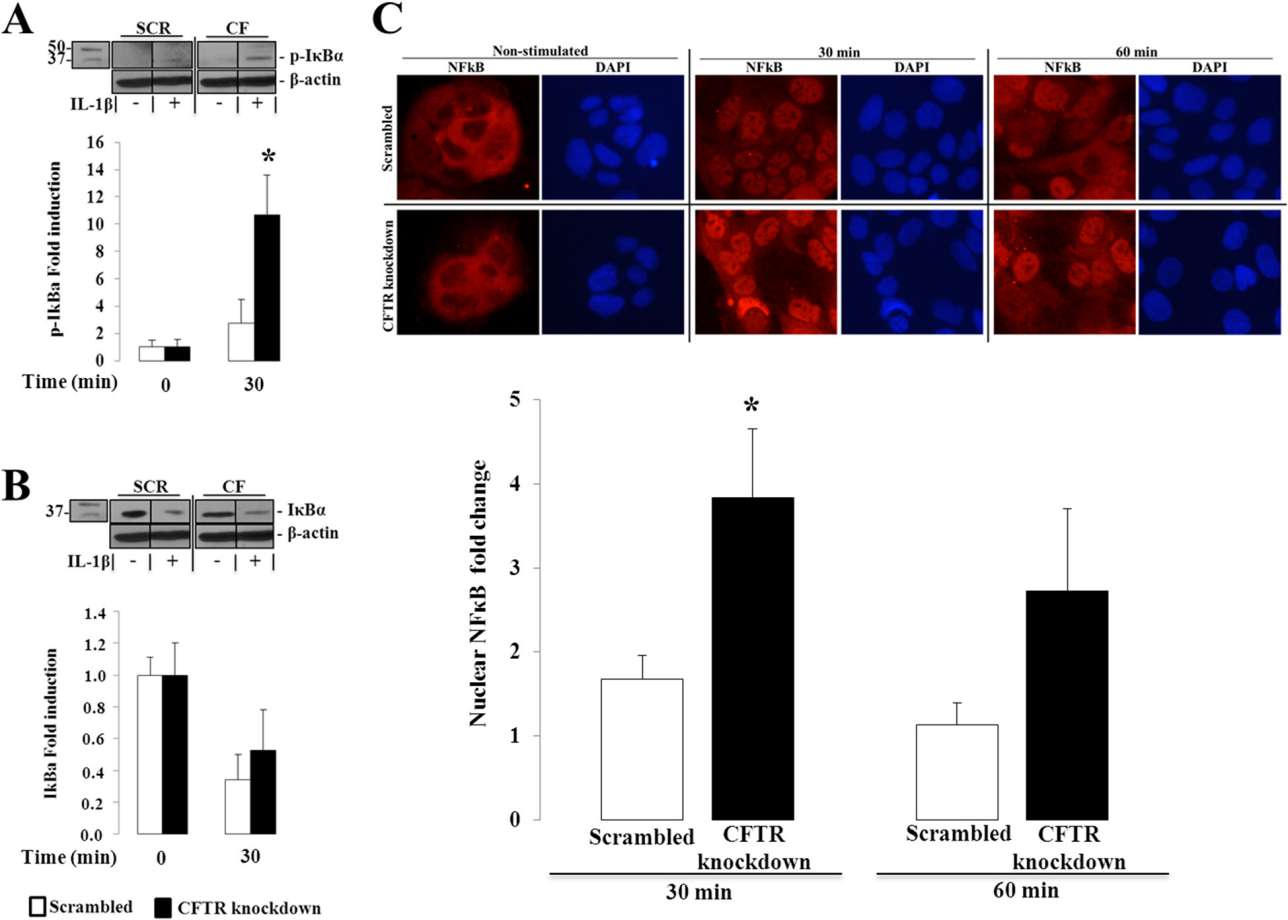
Effect of CFTR knockdown on IκB phosphorylation and NF-κBp65 nuclear translocation in Caco-2/15 cells. Cells were incubated from 0 to 60 min with 25 ng/mL of IL1-β. (**a**) Protein expression of phosphorylated IκB and (**b**) total IκB was analyzed by Western blotting. Data represent the means ± SEM of *n* = 4 independent experiments and both protein expressions were normalized to β-actin and reported as fold induction over basal expression. Western blot images are representative of the results obtained in the four independent cell culture experiments. (**c**) Ratio of nuclear to cytoplasmic fluorescence intensity of NF-κBp65 was calculated following the quantitative analysis of digitized immunofluorescence images. Data represent the means ± SEM of *n* = 5 independent experiments. Data are reported as fold induction over basal ratio of nuclear to cytoplasmic fluorescence intensity of NF-κB p65 after a 30 and 60 min-incubation with 25 ng/ml of IL1-β. Immunofluorescence images representing NF-κBp65 nuclear and cytoplasmic fluorescence intensity (in red) and DAPI nuclear staining (in blue) from scrambled and CFTR knockdown cells after 0, 30 and 60 min of IL1-β stimulation. Contrast and luminosity were standardised for all images. *p < 0.05 vs. scrambled-infected cells

NF-κB is a key transcription factor important in the regulation of cytokine-induced IL-8 transcription. The initial steps for NF-κB activation involve IκBα phosphorylation, ubiquitination and subsequent proteasomal destruction, which release NF-κB and allow its translocation across the nuclear membrane [19]. We therefore determined whether phosphorylation of IκBα and nuclear translocation of the NF-κB p65 subunit were influenced by CFTR gene depletion following IL-1β treatment. As expected, addition of IL-1β resulted in the phosphorylation of IκBα and a trend toward a reduced expression of total IκBα, suggesting proteasomal degradation (Fig. 5a-b). Levels of phosphorylated IκBα increased by more than 10-fold in knockdown cells whereas scrambled cells exhibited a 2.8-fold increase.

Immunofluorescence analysis revealed that the mean ratio of nuclear to cytoplasmic fluorescence intensity did not differ significantly between CFTR knockdown and scrambled cells at baseline (0.446 ± 0.086 vs. 0.793 ± 0.240, *p* = 0.211). However, the knockdown cells exhibited a 4- and 3-fold increase in this ratio after 30 and 60 min of exposure to IL-1β, respectively. In comparison, scrambled cells treated with IL-1β displayed a 1.6 and 1-fold increase of this ratio at the same time points (Fig. 5c).

### HT-29 cells

**Fig. 6:**
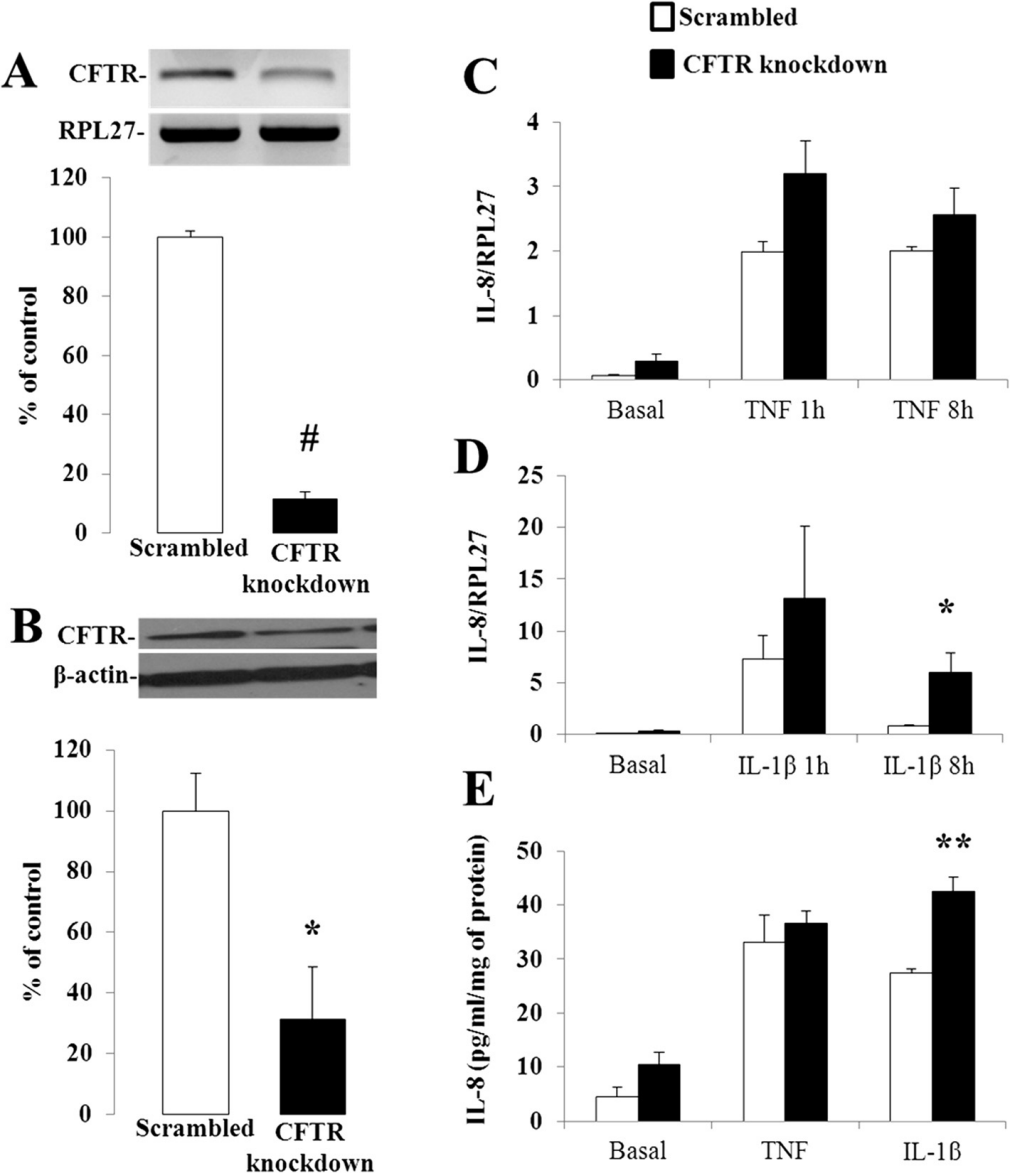
Effect of CFTR knockdown on IL-8 mRNA and secretion levels in HT-29 cells. HT-29 cells were infected with a lentiviral vector carrying either a scrambled sequence or shRNAi against CFTR and analyzed for gene (**a**) and protein (**b**) expression of CFTR by Q-PCR and Western blotting. Results represent the means ± SEM of three independent experiments and are illustrated as % of controls after calculating the data as densitometric ratios of CFTR to the housekeeping gene RPL27 for gene expression (*n* = 3) or CFTR to β-actin for protein expression (*n* = 3). CFTR knockdown or control (Scrambled) cells were incubated 1 and 8 h in the absence or presence of 10 ng/mL of either TNF (**c**) or IL1-β (**d**). Total RNA was collected and subjected to Q-PCR using intron-spanning primers for the IL-8 gene. (**e**) CFTR knockdown or control (Scrambled) cells were incubated 24 h in the absence or presence of 10 ng/mL of either TNF or IL1-β and supernatants were collected to quantify the secretion of IL-8 by ELISA. Results represent the means ± SEM of *n* = 3 independent experiments and are illustrated as pg/mL normalized to total protein concentration. *p <0.05, **p <0.01 and #*p* <0.0001 vs. scrambled-infected cells

Further evidence of the pro-inflammatory effect of CFTR knockdown in intestinal cells was obtained using a different cell line (i.e. HT-29), which displays marked features of human intestinal epithelial cells and express CFTR. Infection of HT-29 cells with a lentiviral vector harbouring shRNAi targeting CFTR resulted in the knockdown of CFTR mRNA and protein by 88 % and 69 % respectively (Fig. 6a-b). Compared to non-infected cells, scrambled infection neither affected CFTR mRNA (100 ± 55.3 vs. 83 ± 37.6, *n* = 3) nor protein expression (100 ± 14.8 vs. 103 ± 13.0 %, *n* = 3). In addition, cell viability remained unaffected whether by lentiviral infection or treatment with TNF and IL-1β (see Additional file 3). We then assessed IL-8 mRNA and secretion levels in HT-29 cells subjected to TNF or IL-1β treatment. We first compared non-infected cells to scrambled cells. Similar to Caco-2/15 cells, scrambled infection did not significantly affect IL-8 mRNA expression (0.026 ± 0.006 vs. 0.069 ± 0.018, *n* = 6) or IL-8 secretion levels compared to non-infected cells (1.98 ± 0.79 vs. 4.59 ± 1.62 pg/ml; *n* = 3). However, HT-29 cells constitutively expressed and secreted more IL-8 and were more highly responsive to proinflammatory cytokines than Caco-2/15 cells. CFTR knockdown of HT-29 cells resulted in a significant 8-fold increase of IL-8 mRNA levels and a 1.5-fold increase in IL-8 secretion in response to IL-1β (Fig. 6d-e). In contrast to Caco-2/15 cells, IL-8 mRNA levels and secretion did not differ between scrambled and CFTR knockdown cells in response to TNF.

### Intestinal histology

**Fig. 7:**
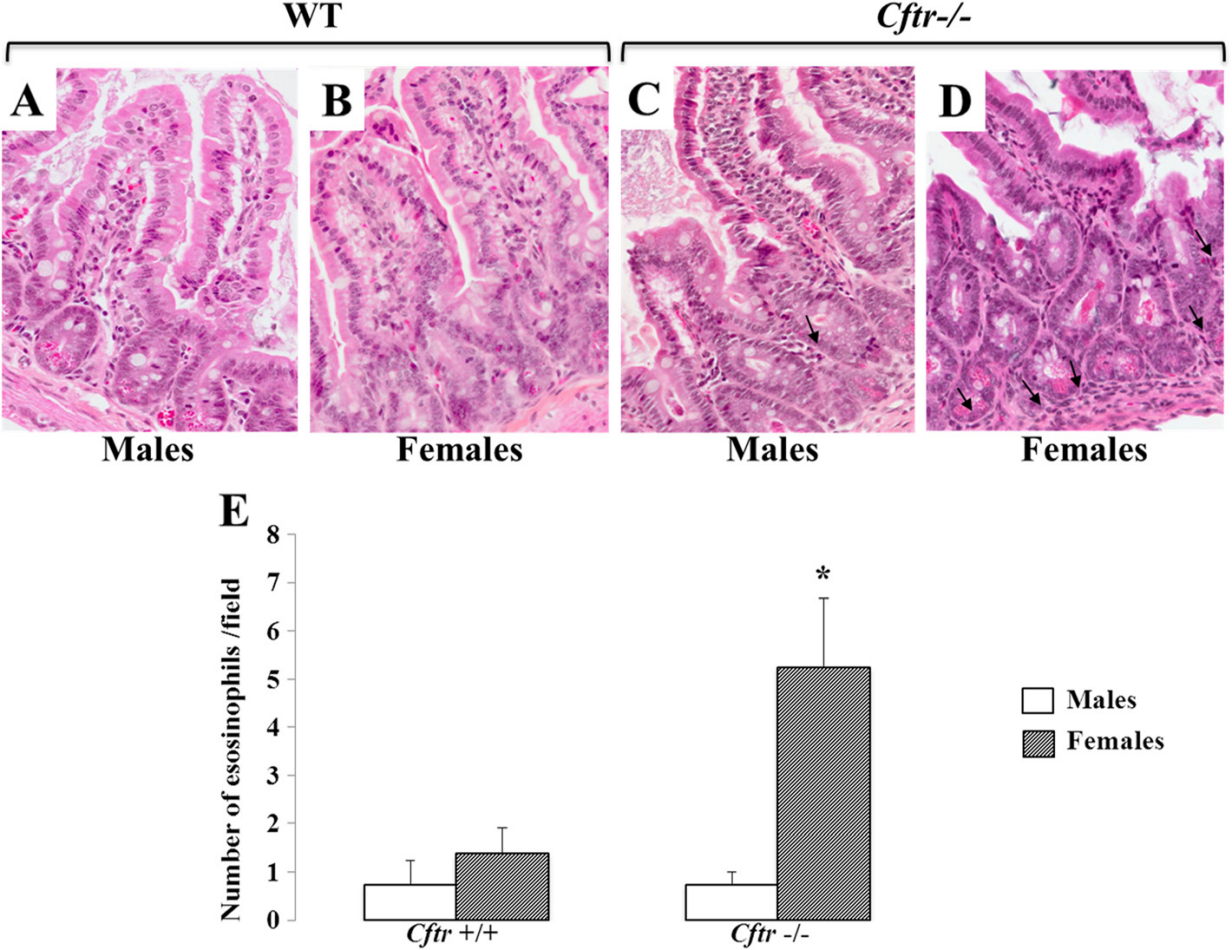
Eosinophil counts in the jejunal mucosa of *Cftr −/−* and *Cftr +/+* mice. Hematoxylin-eosin staining of female mice jejunum showed the presence of eosinophils infiltrating the mucosa in the *Cftr−/−* specimen (**d**) compared to the *Cftr+/+*jejunum (**b**). In contrast, no such difference is observed in the male specimens (**a** and **c**). Black arrows indicate the presence of eosinophils. (**e**) Quantitation of eosinophils in the intercryptic lamina propria of *Cftr−/−* and *Cftr+/+* mice from both genders. Results represent the means ± SEM of *n* = 4 and 5 *Cftr+/+* males and females respectively, and 4 and 4 *Cftr−/−* males and females. 400 × magnification. *p <0.03 vs. *Cftr+/+* females

IL-8 secretion represents an important primary event for the activation of immune and inflammatory cells in the intestinal mucosa in response to various insults. To determine whether our results could be transposed *in vivo*, we quantified the number of eosinophils in the intestinal mucosa of *Cftr* knockout mice to that of mice with intact *Cftr* of the same age and receiving the same diet*.* As shown in Fig. 7, we found a greater number of infiltrating eosinophils in the jejunal mucosa of *Cftr −/−* females, with roughly four times more eosinophils than *Cftr +/+* females (5.3 ± 1.4 vs. 1.4 ± 0.5; *p* <0.03). Interestingly, the counts of eosinophils in the jejunal mucosa of *Cftr−/−* and *Cftr+/+* males were lower and did not differ significantly (0.8 ± 0.5 vs. 0.8 ± 0.3).

## Discussion

CF lungs and airways exhibited a proinflammatory phenotype. However, more recently, exaggerated inflammation has also been documented in other cell types thereby suggesting that hyperinflammatory responses are not exclusively restricted to airway and lung epithelial cells. Indeed, inflammation has been documented in the pancreas and intestine, two major CF-affected tissues [3, 11]. Intestinal inflammation is present in both *Cftr* knockout mice [4] and patients [5, 11, 12] and may, in fact, account for the 1.7-fold increase in the incidence of inflammatory bowel disease seen in CF patients [10]. CF patients also develop digestive tract cancers at a younger age and at a higher rate than non-CF individuals [20]. Whether chronic intestinal inflammation is associated with the development of these cancers, as observed in other chronic intestinal inflammatory disorders such as Crohn’s disease, is still debatable [21]. In addition, it has been speculated that chronic intestinal inflammation can adversely affect gastrointestinal motility by promoting muscularis overgrowth and submucosal fibrosis, thereby predisposing CF patients to distal ileal obstruction syndrome (DIOS) later in life [22]. Clearly, intestinal inflammation has health implications for these patients. However, the pathogenic mechanisms underlying this inflammation remain relatively unexplored. In light of the evidence that signs of intestinal inflammation may be present in the sterile bowel of CF foetuses combined with *in vitro* data showing that the lack of CFTR was intrinsically associated with exaggerated inflammation in cells such as macrophages and dendritic cells [6, 23, 24], we postulated that genetic depletion of CFTR will affect the inflammatory status of intestinal epithelial cells.

To test this hypothesis, we used two enterocyte cell models, Caco-2/15 and HT-29, in which we altered CFTR gene and protein expression. As opposed to airway and lung cells, there are no CF intestinal cell lines available for these types of studies. Furthermore, intestinal explants from patients or primary human intestinal epithelial cells display very limited *in vitro* viability, which greatly hampers long-term investigations. Human intestinal cell lines therefore represent a good available cell culture model as they retain the capacity to spontaneously differentiate into cells possessing the morphology and functions of human enterocytes [25]. This study added Caco-2/15 and HT-29 cells to the growing list of cells exhibiting exaggerated inflammatory responses when CFTR is either absent, mutated or knocked down. We showed that as little as a 39 % CFTR protein reduction leads to an increased basal and stimulated secretion of the pro-inflammatory chemokine, IL-8, irrespective of the initiating cytokine, thereby suggesting that downstream effectors common to IL-1β, TNF and IL-8 are affected by CFTR depletion. The fact that we were able to confirm and extend those findings to a different intestinal cell line further strengthens our hypothesis. The release of the proinflammatory cytokine IL-6 was several fold lower than IL-8 and was induced only in knockdown cells after IL-1β treatment. The observation that IL-1β elicited such a marked increase in IL-6 production in knockdown cells provides additional evidence that decreased CFTR expression alters the cellular immune response. It has been previously shown that TNF was less potent than IL-1β in inducing IL-6 secretion in Caco-2 cells [26], which might explain why TNF did not stimulate IL-6 production in our cells. Such findings cannot be attributed to cytotoxic and apoptotic effects, as cell viability remained above 90 % and levels of Bax, a pro-apoptotic protein, remained unchanged in all experimental conditions. Our results corroborate other studies performed in various epithelial and non-epithelial cells indicating that gene deletion or knockdown of CFTR confers a propensity to exaggerated inflammatory responses to bacterial and cytokine but not viral stimuli [18, 23, 27–29]. This phenotype was, however, partially restored by pharmacological (e.g. forskolin and IBMX) or genetic (e.g. transfection with wt-CFTR) means [30, 31]. Interestingly, we also observed that forskolin/IBMX treatment of Caco-2/15 cells resulted in a significant reduction in IL-1β and, to a lesser extent, TNF-induced IL-8 release.

The secretion of IL-8 is in part regulated transcriptionally by several transcription factors and post-transcriptionally through the stabilization of mRNA [16]. As a result of transcriptional repression and mRNA destabilization, virtually no IL-8 synthesis is present in basal conditions. The fact that we observed nearly a two-fold increase in basal IL-8 concentration of knockdown cells may indicate that these regulatory processes are altered when CFTR expression is reduced. In fact, the higher levels of IL-8 in the supernatant of cells knocked down for CFTR is likely attributable to an increase in its gene expression as mRNA steady-state levels were markedly enhanced in these cells in response to IL-1β and TNF. Conversely, IL-8 mRNA stability was found to be unaltered by CFTR depletion. This observation is in contrast with previous studies reporting increased IL-8 mRNA stabilization in lung and bronchial CF cell lines as well as in primary cultures [27, 32], suggesting that the molecular mechanisms governing IL-8 mRNA stability may be cell-type specific. It has been suggested that constitutive activation of MAPK pathways may promote IL-8 mRNA stabilization [27]. The absence of any difference in IL-1β-induced phosphorylation of p38 and JNK MAPK between CFTR knockdown and scrambled cells may be an alternative explanation for the lack of effect on mRNA stability. Nevertheless, the involvement of these pathways cannot be completely ruled out as we observed that the p38MAPK inhibitor, BIRB796, more effectively reduced IL8 secretion in scrambled than CFTR knockdown cells, possibly suggesting a greater activation of p38MAPK when CFTR expression is reduced (unpublished data). Conversely, the level of phosphorylation of ERK, another class of MAPKs, appears to be modulated by CFTR gene depletion. Indeed, phosphorylation of ERK was significantly enhanced when CFTR was knocked down from Caco-2/15 cells. Likewise, ERK phosphorylation has been shown to be upregulated in other CF models [17, 18]. As opposed to JNK and p38 pathways, whose role in IL-8 regulation is undeniable, information about the involvement of ERK pathway is more limited. However, studies on ERK inhibitors or constitutively active mutants in other cell models have suggested that this pathway may contribute to IL-8 expression in conjunction with NF-κB [33] or through regulation of IL-8 mRNA stability [27]. Likewise, we found that phosphorylation of IκBα and nuclear translocation of NF-κB in response to IL-1β was significantly enhanced in the knockdown cells. NF-κB is normally retained in the cytoplasm as an inactive complex with the inhibitory proteins, IκB. In response to proinflammatory stimuli, the IkBs are rapidly phosphorylated and degraded thus freeing NF-κB subunits to translocate into the nucleus. Nuclear import is critical for NF-κB transcriptional activity and IL-8 promoter contains an NF-κB -binding site that is required for activation of its transcription. Indeed, we observed IL-8 mRNA levels to be induced by pro-inflammatory stimuli in our cells. Thus, one may speculate that the increased Il-8 gene and secreted protein levels seen in CFTR knockdown intestinal cells in response to IL-1β may be directly related to NF-κB activation.

Interestingly, we did not detect any difference in the secretion of the anti-inflammatory IL-10 between the control and knockdown cells in response to pro-inflammatory stimuli. While IL-10 secretion parallels that of IL-8 and IL-6, one would have expected to see a greater release in the knockdown cells. These data suggest that CFTR knockdown cells display blunted anti-inflammatory responses when challenged with proinflammatory cytokines, perhaps contributing to the exaggerated secretion of IL-8 and IL-6 and the deregulated local inflammatory response. The finding that IL-10 deficient mice develop spontaneous chronic intestinal inflammation has established a key role for IL-10 in intestinal immune homeostasis [34]. Indeed, lamina propria mononuclear cells isolated from inflamed mucosa collected from patients with Crohn’s disease and ulcerative colitis produced less IL-10 [35]. To our knowledge, IL-10 has never been investigated in the context of CF-intestinal inflammation; however, decreased IL-10 production has been reported in CF airways whereby it was postulated that IL-10 deficiency may contribute to the pathogenesis of CF lung disease [36–38]. One can speculate that a reduced expression of CFTR may result in an imbalance between pro-inflammatory and anti-inflammatory mediators that impedes the resolution of inflammation and leads to the perpetuation of a chronic inflammatory state.

Epithelial cells form an interface between pathogenic microorganisms and host tissues and, in the intestine, represent the first host cells to come in contact with enteric pathogens. Upon bacterial entry or pro-inflammatory cytokine exposure, intestinal epithelial cells, both primary and cell lines, secrete IL-8, a known chemokine, whose most important functions are to attract and activate leucocytes [39, 40]. The recent demonstration of intestinal dysbiosis, with a shift towards the abundance of potentially harmful species, in homozygous ΔF508 and severe CF patients further lends support to the physiological implication of our findings [41]. It is suggested that following a breach in the barrier function, the commensal bacteria gain access to the mucosa, thereby leading to inflammatory responses, which, in the case of CF, are deregulated. These findings coupled with the fact that CF intestinal mucosa exhibits increased neutrophil, eosinophil and lymphocyte infiltration [22], point to a contributing role of enterocytes in the onset, development or maintenance of CF intestinal inflammation. Moreover, the fact that Caco-2/15 and HT-29 cells responded to IL-1β and TNF, two cytokines that are predominantly released by activated macrophages during inflammatory responses, implies that intestinal epithelial cells may serve to amplify and sustain an ongoing immune process. In line with this hypothesis and previous studies [4, 42], our *in vivo* results indeed showed a greater number of infiltrating eosinophils in the intestinal mucosa of *Cftr−/−* mice without evidence of intestinal obstruction compared to age-matched controls receiving the same feeding and drinking regimen. To our knowledge, we are the first to report a sex difference in the severity of intestinal inflammation with *Cftr−/−* females being more affected than *Cftr−/−* males. This observation is not surprising given the presence of a CF gender gap whereby the female gender is associated with a worse prognosis. The role of estrogens in the regulation of inflammatory processes has been primarily studied in the context of CF lung infection. Administration of estrogens to CF mice prior to *Pseudomonas Aeruginosa* infection has caused an increased number of white blood cells and neutrophils in the lungs and in the brochoalveolar lavage [43].

The exact molecular mechanisms linking loss of functional CFTR and exacerbated inflammatory responses are still minimally understood. It has been documented that CFTR tail binds to multiple proteins including protein kinases involved in inflammation signalling. Kunzelmann and Mehta have recently put forward the interesting notion that CFTR acts as a hub, which may differ in its composition between cell types and explain the heterogeneity of cell responses to the loss of functional CFTR [44]. The authors suggested that this hub confers upon cells the ability to cope with external challenges. Disruption of its structure, for instance in case of loss or mutations of CFTR, may thus suppress or alter the optimal response to cell challenge. The fact that moderate protein knockdown disrupts this function suggests that there is a minimal tissue-specific requirement for normal CFTR function. Our observations were made on undifferentiated, and thus non-polarized, cells, which could represent a major caveat. However, our findings demonstrate that the presence of cellular CFTR, rather than its apical localization, is a key feature for its anti-inflammatory properties. Nevertheless, given that we failed to chronically inhibit CFTR of Caco-2/15 cells by pharmacological means, we cannot exclude the possibility that alterations in CFTR-mediated chloride transport may also lead to hyperinflammatory responses. The fact that cytokine-stimulated IL-8 secretion was markedly blunted by forskolin and IBMX strengthens this possibility.

It remains difficult to ascribe inflammatory responses unambiguously to defective CFTR as there are nearly 2000 mutations(http://www.genet.sickkids.on.ca/app) that have been documented in the CFTR gene, and which influence the amount and function of CFTR in their own respective manner. Thus far, only one study has reported the lack of correlation between CFTR mutations and enteroscopy findings in a cohort of CF patients [12]. The absence of association may have been due to insufficient statistical power as only 41 patients were assessed in this study. The residual expression of CFTR in our knockdown cells makes data extrapolation to CF patients carrying severe CFTR genotypes difficult. Nevertheless, they can bring to light the situation occurring either in individuals with milder CFTR mutations characterized by reduced CFTR biosynthesis or ion conductance, or in CFTR heterozygous carriers who display CF-related manifestations with a strong inflammatory component such as asthma, sinusitis and chronic pancreatitis [45, 46].

## Conclusion

In summary, we report that CFTR gene and protein depletion of intestinal Caco-2/15 and HT-29 cells lead to increased IL-6 and −8 secretion, which originates in part from a greater activation of ERK MAPK and NF-κB pathways coupled to a blunted IL-10 secretion. Our study provides novel data arguing in favour of a contributing role of CFTR in the pathogenesis of CF-related intestinal inflammation. Altogether, these findings provide support to the theory that CFTR disruption, even moderate, may prevent cells from optimally responding to exogenous or endogenous challenges that trigger an inflammatory response. Such observations are of particular interest for CF patients who were found to display alterations in their intestinal microbiota, thus predisposing them to pathogens that may elicit exaggerated inflammatory responses [47]. The emergence of CF intestinal organoid culture system will likely serve as a valuable tool for the further pathogenic characterization of CF-related intestinal inflammation [48].

## Additional files

Additional file 1:
**Cell viability of Caco-2/15 cells exposed to the various experimental conditions: Cell viability of Caco-2/15 cells was assessed with the Trypan blue exclusion method.** All treatments were 24 h in duration and the dose of TNF and IL-1β used was 25 ng/mL. Results are indicated as percentages. Data represent the means ± SEM of three experiments. #*p* < 0.05 vs. non-infected cells. (PDF 77 kb) Additional file 2:
**Bax protein expression in Caco-2/15 cells exposed to the various experimental conditions.** Caco-2/15 cells infected or not were stimulated 24 h with either TNF or IL-1β at 25 ng/ml. Bax protein expression was analyzed by Western blotting. Data represent the means ± SEM of *n* = 3 independent experiments and are reported as the Bax/β-actin ratio. (TIFF 227 kb) Additional file 3:
**Cell viability of HT-29 cells exposed to the various experimental conditions.** Cell viability of HT-29 cells was assessed with the Trypan blue exclusion method. All treatments were 24 h in duration and the dose of TNF and IL-1β used was 10 ng/ml. Results are indicated as percentages. Data represent the means ± SEM of three experiments. Results are non-significant. (PDF 76 kb)

## Abbreviations

CF: Cystic fibrosis
CFTR: Cystic fibrosis transmembrane conductance regulator
ERK: Extracellular signal-regulated kinases
FBS: Fetal bovine serum
IBMX: 3-Isobutyl-1-methylxanthin
IL-1β: Interleukin-1β
IL-8: Interleukin-8
JNK: Jun N-terminal kinases
MAPK: Mitogen-activated protein kinase
MPK-1: Mitogen-activated protein kinase phosphatase-1
NEAA: Non-essential amino acids
NF-κB: Nuclear factor-kappa B
PS: Penicillin-streptomycin
shRNAi: Short hairpin RNA interference
TNF: Tumor necrosis factor
