# Supplementary material for: CFTR Knockdown induces proinflammatory changes in intestinal epithelial cells
Source: J Inflamm (Lond). 2015 Nov 7;12:62. doi: 10.1186/s12950-015-0107-y (PMC4636765; doi:10.1186/s12950-015-0107-y)
Supplement: Additional file 1: — Cell viability of Caco-2/15 cells exposed to the various experimental conditions: Cell viability of Caco-2/15 cells was assessed with the Trypan blue exclusion method. All treatments were 24 h in duration and the dose of TNF and IL-1β used was 25 ng/mL. Results are indicated as percentages. Data represent the means ± SEM of three experiments. #p < 0.05 vs. non-infected cells. (PDF 77 kb) [file 12950_2015_107_MOESM1_ESM.pdf]

Additional file 1. Cell viability of Caco-2/15 cells exposed to the various experimental conditions

Experimental conditions	Untreated	Treated with pro-inflammatory agents	
		TNF-α	IL-1β
**Non-infected**	96±0.53	95±1.50	92±1.40
**Scrambled**	94±1.49	95±1.00	95±0.58
**CFTR knockdown**	95±1.18	93±2.99	96±0.15
**Forskolin/IBMX**	81±2.3#	89±0.26	91±2.11

Cell viability of Caco-2/15 cells was assessed with the Trypan blue exclusion method. All treatments were 24 h in duration and the dose of TNF and IL-1β used was 25 ng/mL. Results are indicated as percentages. Data represent the means±SEM of three experiments. #$p<0.05$ vs. non-infected cells.
