# Supplementary figures and images for: CFTR Knockdown induces proinflammatory changes in intestinal epithelial cells
Source: J Inflamm (Lond). 2015 Nov 7;12:62. doi: 10.1186/s12950-015-0107-y (PMC4636765; doi:10.1186/s12950-015-0107-y)

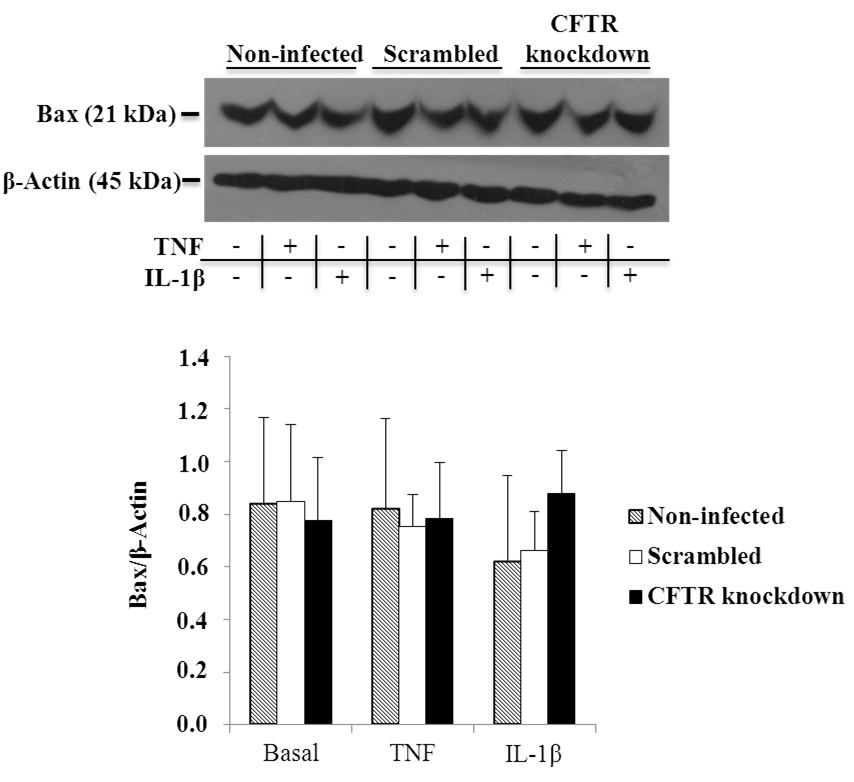

Supplement: Additional file 2: — Bax protein expression in Caco-2/15 cells exposed to the various experimental conditions. Caco-2/15 cells infected or not were stimulated 24 h with either TNF or IL-1β at 25 ng/ml. Bax protein expression was analyzed by Western blotting. Data represent the means ± SEM of n = 3 independent experiments and are reported as the Bax/β-actin ratio. (TIFF 227 kb) [file 12950_2015_107_MOESM2_ESM.tiff]
