# Supplementary material for: CFTR Knockdown induces proinflammatory changes in intestinal epithelial cells
Source: J Inflamm (Lond). 2015 Nov 7;12:62. doi: 10.1186/s12950-015-0107-y (PMC4636765; doi:10.1186/s12950-015-0107-y)
Supplement: Additional file 3: — Cell viability of HT-29 cells exposed to the various experimental conditions. Cell viability of HT-29 cells was assessed with the Trypan blue exclusion method. All treatments were 24 h in duration and the dose of TNF and IL-1β used was 10 ng/ml. Results are indicated as percentages. Data represent the means ± SEM of three experiments. Results are non-significant. (PDF 76 kb) [file 12950_2015_107_MOESM3_ESM.pdf]

Additional file 3. Cell viability of HT-29 cells exposed to the various experimental conditions

Experimental conditions	Untreated	Treated with pro-inflammatory agents	
		TNF-α	IL-1β
**Non-infected**	93±3.03	90±3.36	94±0.32
**Scrambled**	96±0.90	93±0.37	92±1.46
**CFTR knockdown**	92±1.81	92±1.23	90±2.87

Cell viability of HT-29 cells was assessed with the Trypan blue exclusion method. All treatments were 24 h in duration and the dose of TNF and IL-1β used was 10ng/ml. Results are indicated as percentages. Data represent the means±SEM of three experiments. Results are non-significant.
